# Design and Testing of Novel Lethal Ovitrap to Reduce Populations of *Aedes* Mosquitoes: Community-Based Participatory Research between Industry, Academia and Communities in Peru and Thailand

**DOI:** 10.1371/journal.pone.0160386

**Published:** 2016-08-17

**Authors:** Valerie A. Paz-Soldan, Josh Yukich, Amara Soonthorndhada, Maziel Giron, Charles S. Apperson, Loganathan Ponnusamy, Coby Schal, Amy C. Morrison, Joseph Keating, Dawn M. Wesson

**Affiliations:** 1 Department of Global Community Health and Behavioral Sciences, Tulane University School of Public Health and Tropical Medicine, 1440 Canal Street, New Orleans, LA 70112 United States of America; 2 Facultad de Salud Pública y Administración, Universidad Peruana Cayetano Heredia, Avenida Honorio Delgado 430, Urb. Ingeniería, San Martín de Porres, Lima, Peru; 3 Center for Applied Malaria Research and Evaluation, Tulane University School of Public Health and Tropical Medicine, 1440 Canal Street, New Orleans, LA 70112, United States of America; 4 Department of Tropical Medicine, Tulane University School of Public Health and Tropical Medicine, 1440 Canal Street, New Orleans, LA 70112, United States of America; 5 Institute for Population and Social Research, Mahidol University, Salaya, Phuthamontol, Nakornpathom, Thailand, 73170; 6 Department of Health Science and Recreation, San Jose State University, 1 Washington Sq, San Jose, CA 95192, United States of America; 7 Department of Entomology and Plant Pathology, North Carolina State University, Raleigh, NC 27695, United States of America; 8 Department of Entomology and Nematology, University of California Davis, One Shields Ave, Davis, CA 95616, United States of America; 9 Vector Borne Infectious Disease Research Center, Tulane University School of Public Health and Tropical Medicine, 1440 Canal Street, New Orleans, LA 70112, United States of America; Institut Pasteur, FRANCE

## Abstract

**Background:**

Dengue virus (and Chikungunya and Zika viruses) is transmitted by *Aedes aegypti* and *Aedes albopictus* mosquitoes and causes considerable human morbidity and mortality. As there is currently no vaccine or chemoprophylaxis to protect people from dengue virus infection, vector control is the only viable option for disease prevention. The purpose of this paper is to illustrate the design and placement process for an attractive lethal ovitrap to reduce vector populations and to describe lessons learned in the development of the trap.

**Methods:**

This study was conducted in 2010 in Iquitos, Peru and Lopburi Province, Thailand and used an iterative community-based participatory approach to adjust design specifications of the trap, based on community members’ perceptions and feedback, entomological findings in the lab, and design and research team observations. Multiple focus group discussions (FGD) were held over a 6 month period, stratified by age, sex and motherhood status, to inform the design process. Trap testing transitioned from the lab to within households.

**Results:**

Through an iterative process of working with specifications from the research team, findings from the laboratory testing, and feedback from FGD, the design team narrowed trap design options from 22 to 6. Comments from the FGD centered on safety for children and pets interacting with traps, durability, maintenance issues, and aesthetics. Testing in the laboratory involved releasing groups of 50 gravid *Ae*. *aegypti* in walk-in rooms and assessing what percentage were caught in traps of different colors, with different trap cover sizes, and placed under lighter or darker locations. Two final trap models were mocked up and tested in homes for a week; one model was the top choice in both Iquitos and Lopburi.

**Discussion:**

The community-based participatory process was essential for the development of novel traps that provided effective vector control, but also met the needs and concerns of community members.

## Background

*Aedes aegypti* and *Aedes albopictus* mosquitoes are the most important vectors for Dengue virus (DENV), Chikungunya virus (CHIKV), Zika virus (ZV) and urban yellow fever (YF), all of which contribute significantly to human morbidity and mortality globally [1–4]. Both mosquito species are highly adapted to human housing where they develop in a large variety of human-made containers, and adults feed on human hosts without the need to leave the location they were born [5–6]. As the first dengue vaccines have been licensed, difficulties with widespread deployment and efficacy indicate that vector control will continue to play a significant role in integrated dengue prevention and control programs, and remains the viable option for other *Aedes* borne diseases that have recently emerged, such as Chikungunya and Zika [7–8]. There is a strong need for new vector control strategies that impact multiple disease systems.

Historically, source reduction (elimination of water holding containers), larvicides (*e*.*g*. temphos, *Bacillus thuringiensis* var. *israelensis*), biological control (e.g. copepods, mosquito fish), and adulticides including space sprays (*e*.*g*. pyrethroids), insecticide treated nets, residual wall and foliage sprays, and lethal traps have all been used for the control of disease vectors [9]. However, several factors have limited the success of these strategies for controlling *Aedes* mosquito populations [9]. Biological factors include resistance to insecticides and adaptive vector behavior; however, logistical concerns of coverage (which needs to be high), frequency of application, and community acceptance have led to a lack of sustainable success to many dengue control programs [10–12]. In the case of lethal traps, though there have been some unsuccessful attempts, it appears that thorough coverage with an appropriate number of traps per unit area is key to success [13].

The attractive baited lethal ovitrap (ALOT) is a new strategy that has shown promise in laboratory settings and has the potential to significantly reduce the *Aedes* mosquito populations. Preliminary results from one year of a large intervention trial conducted in Iquitos, Peru where nearly 8,000 ALOT traps were deployed in a core area (450 households) monitored for dengue disease and transmission and 2,000 surrounding buffer households showed a 75% reduction in dengue incidence in the areas with traps compared to a similar control neighborhood with no traps [14]. Entomological survey data from the same study demonstrated a reduction in older egg-laying mosquitoes in the neighborhood with traps [14]. Furthermore, ALOT traps continued to be used by the Peruvian Ministry of Health in smaller Amazonian Cities and in Lima after the trial (ACM personal communication). The ALOT is designed to reduce the number of mosquitoes through three main actions: 1) Attraction: *Aedes aegypti* are attracted to odors emitted by a specially formulated mix of bacteria species in the water inside the trap [15], as well as visually to the physical design and color of the trap; 2) Adulticide: the inside walls of the trap are lined with a long-lasting insecticide net (LLIN) treated with α-cypermethrin that kills resting adults; and 3) Larvicide: the water inside the trap contains spinosad, designed to kill larvae that might hatch from eggs oviposited by the attracted female mosquitoes.

Importantly, the effectiveness of the ALOT depends on its inherent efficacy (based on its attractiveness to *Aedes* and efficacies of the design and active ingredients) and its acceptance and usability within specific targeted communities. Because details of trap design (*e*.*g*. size, shape, and color) and its placement could vary to some extent based on preferences of community members, the design process should integrate multiple perspectives, including the community, research and industry. Our goal was to develop an attractive lethal trap that would be acceptable to people, engage industry in the process, deploy this new approach under real-life conditions, and document the efficacy and effectiveness of this intervention. As a first step, the purpose of this paper is two-fold: 1) to describe the design and placement process for the ALOT, and 2) to describe lessons learned through the use of this integrated and iterative participatory approach.

## Methods

Institutional Review Board (IRB) approval for this study was obtained from Tulane University School of Public Health and Tropical Medicine in the United States (153793–9), and Mahidol University in Thailand (MU IRB 2010–002.0401). Peruvian local health authorities (Dirección Regional de Salud de Loreto) also provided approval for this study (Oficio #473-2010-GRL-DRSL/30.09.01). As approved by the IRB committees, written consent was requested from community members who participated in the focus group discussions and who piloted the traps in their homes as part of this design and placement phase of the ALOT development.

### Study Areas

Iquitos is a city of approximately 400,000 people located in the Amazon Basin of northeastern Peru, Department of Loreto and has been the site of ongoing studies on dengue epidemiology and *Ae*. *aegypti* ecology [16]. The population works in a range of occupations, including the extractive (*e*.*g*. logging, oil) and agricultural industries. Dengue has been endemic in this region since its re-emergence in the 1990s, and all four dengue serotypes have now circulated—usually only one serotype is dominant at a given point in time [17–19]. Two adjacent neighborhoods, Maynas (MY) and San Antonio (SA) located in the Northern district of Punchana (population ~69,500 in 2007 census) were selected for the larger study (*i*.*e*. testing efficacy of the ALOT) because of their similarities in housing structure and historical levels of *Ae*. *aegypti* infestation and dengue transmission rates [20, 21]. In the efficacy study (to be reported in subsequent papers), the SA neighborhood was designated as the area for trap installation (hereafter treatment area), whereas the MY neighborhood was selected as the control area. All data collection in Iquitos (focus group discussions [FGD], placement of mock traps in homes) occurred in the SA neighborhood.

Lopburi province (~population 730,000) is located in the Central Plain of Thailand [22]. In 2010, the incidence rate for dengue was 5.49 per 100,000 in Lopburi [23]. Within Lopburi, two villages with high incidence rates of dengue were selected as the research sites for this qualitative phase of the study. All data were collected in 2010.

### Study Design

**Fig 1:**
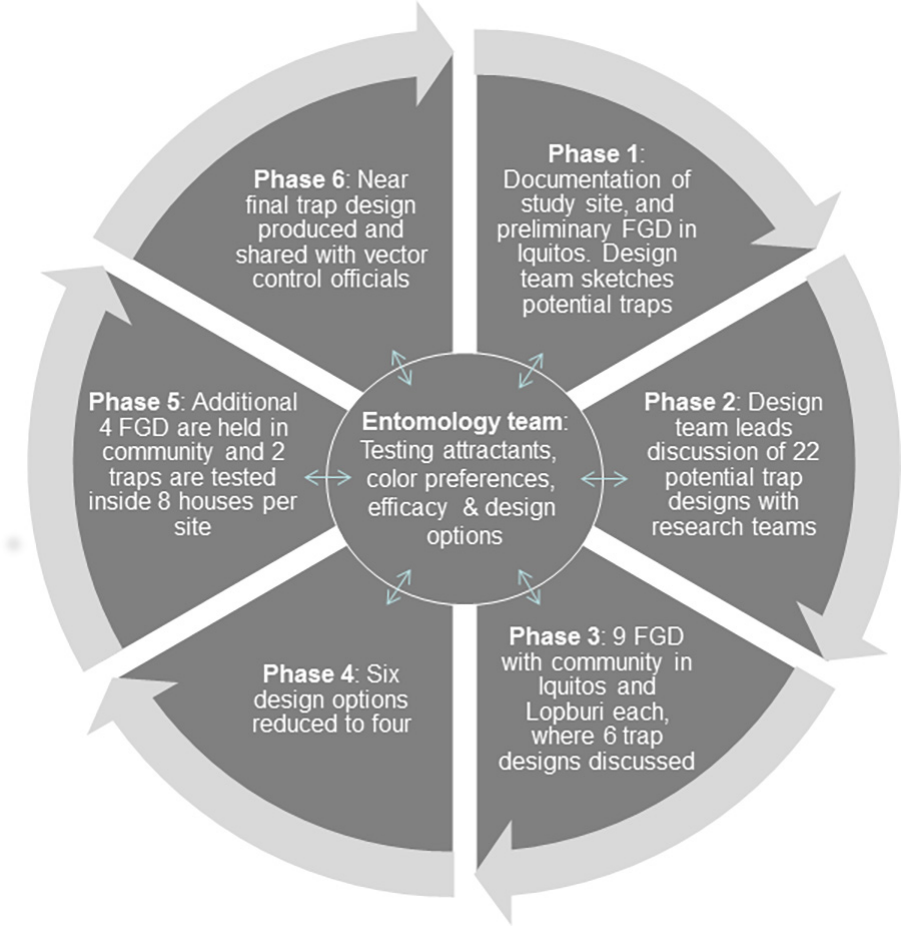
Participatory and interactive model depicting design approach. Model depicting the iterative participatory approach throughout the six phases of the trap design process.

Prior to setting up the efficacy study in Peru and Thailand, efficacy testing for the ALOT was conducted in backyards around the city of New Orleans, Louisiana in the USA [24] because *Aedes aegypti* and *Aedes albopictus* are abundant there. In Peru and Thailand, an iterative participatory approach was used to adjust the specifications of the trap design, based on community perceptions and feedback during discussions, and findings from the entomological lab testing of different trap prototypes, colors, and chemical attractants. Multiple institutions and entities were involved in this participatory process, including researchers (*i*.*e*. a research team consisting of entomologists, epidemiologists, and social scientists), community members, industry (*i*.*e*. design engineers), and ministry of health personnel. Fig 1 illustrates the steps in the design process used to develop and deploy the final trap prototype. Below we describe the six phases of the iterative community-based participatory approach.

Phase 1 focused on documentation of the two study areas via pictures of community features and typical houses (both inside and outside) where the traps would be placed. The photographs were studied by the design team members, as they were the least familiar with characteristics of the study sites. Additionally, using purposive sampling [25] in the SA neighborhoods where the future efficacy study was to be conducted, the research team in Iquitos recruited community members (n = 51) to participate in five initial focus group discussions (FGD); these discussions were held to collect data on human preferences for what residents felt were desirable characteristics of an ALOT in terms of its size and color, to determine where in a home the traps would likely be placed, as well as to identify concerns community members might have with ALOT safety and maintenance. To ensure participation of members of specific demographic groups, FGD were stratified by age group (2 FGD with 25–40 year old members, 2 FGD with 41–65 years old; and 1 FGD with women 25–40 years old who were mothers of children under 5 years old), and by gender (3 FGD with women, 2 FGD with men). The facilitator, an experienced Peruvian social scientist who has been working in Iquitos for many years, led the discussion using a FGD guide she developed in conjunction with the design team; detailed notes were taken by a local notetaker. A summary of the issues discussed regarding potential ALOT components, characteristics and safety was generated and shared with research and design team members. Due to time constraints, phase 1 was only carried out in Iquitos, Peru.

During phase 2, the design team developed and presented the research team with 22 trap design options in sketch format via a video conference call. Each trap design option and its various features were discussed one at a time. The research team participating in the discussion included entomologists in New Orleans, LA and Raleigh, NC, USA; social scientists, epidemiologists, and entomologists in Peru and Thailand; and the design team in Chicago, IL, USA. The social scientist that conducted the FGD in Iquitos also participated, ensuring that items discussed with community members were represented. The discussion culminated in a list of trap features that were considered appealing, a list of essential trap components, and potential challenges.

**Fig 2:**
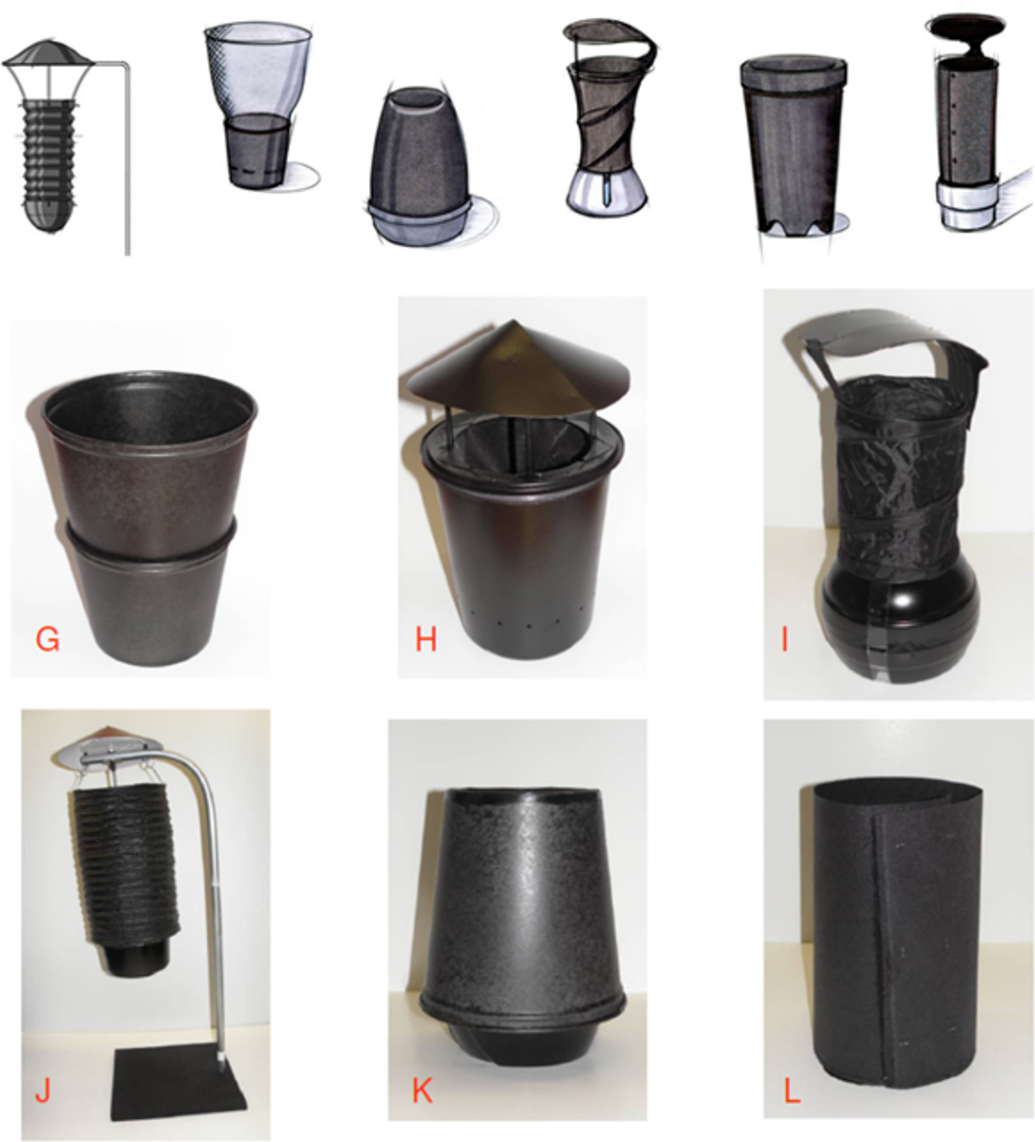
Depiction of second round of trap prototypes. Six trap prototypes presented and discussed in nine focus group discussions in Peru and Thailand.

In phase 3, the design team focused on reducing the number of trap design options from 22 to 6; these six trap designs incorporated features preferred by the research teams (i.e., entomology, epidemiology, and social sciences) and community (Fig 2). These six trap sketches were then presented to and discussed with the community via a series of 9 FGD (n = 81 total participants) in Iquitos and 9 FGD (n = 90 total participants) in Lopburi. Convenience sampling was again used to recruit community members at both sites, with a focus on selecting household decision-makers and community members from a range of ages, gender, and occupations. The composition included 4 FGD with 25–39 year olds (2 all male and 2 all-female), 3 FGD with 40–60 year olds (1 all male and 2 all-female), and 2 FGD with 25–45 year old mothers. Approximately 81 community members participated in Iquitos and 90 participated in Lopburi. FGD participants viewed the 6 sketches of potential traps and discussed features perceived as advantageous or disadvantageous. The discussions culminated in a list of recommended changes, perceived challenges, and a rank of each trap. FGD results were then discussed with the research and design teams.

**Fig 3:**
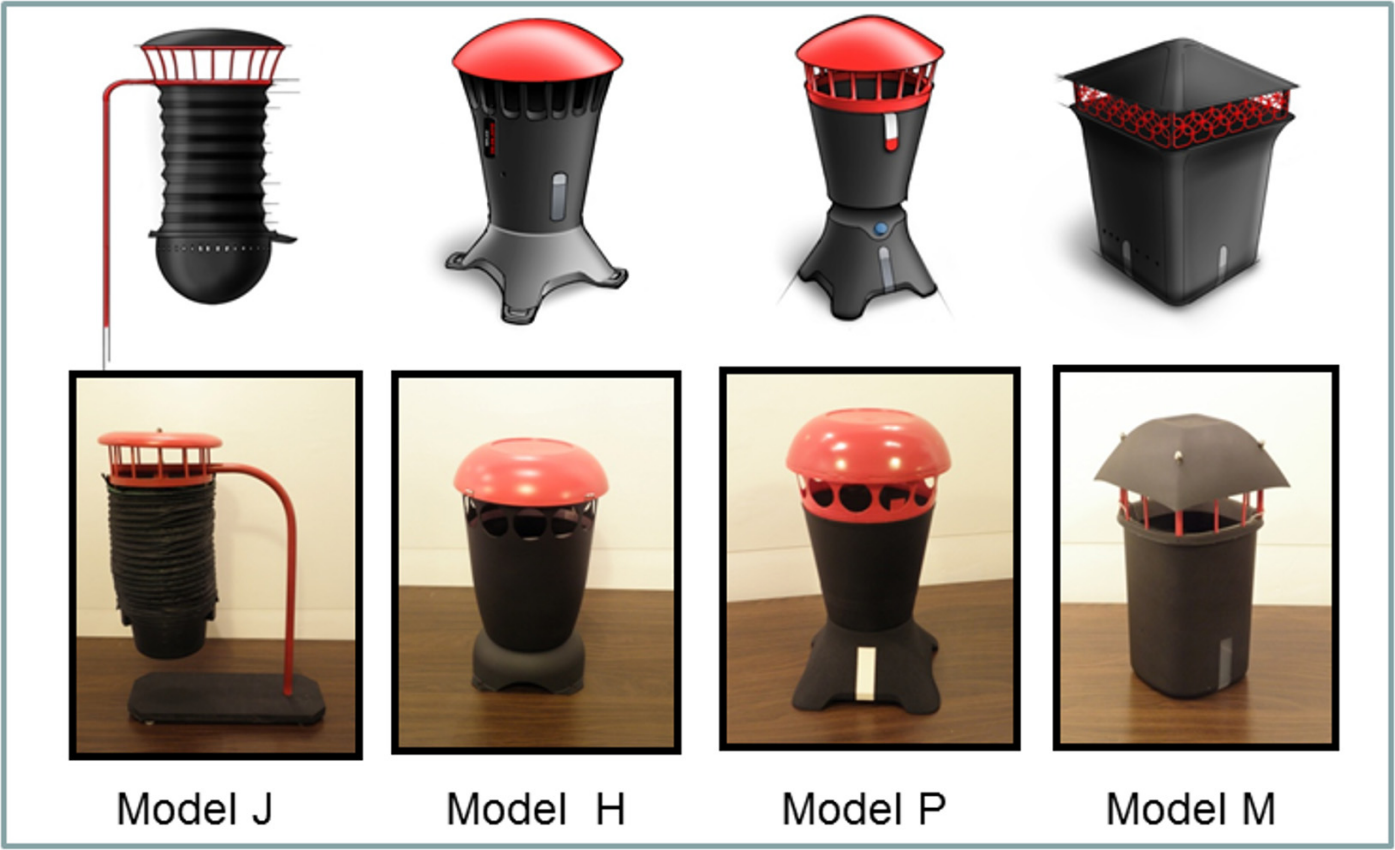
Depiction of third round of trap prototypes. Four trap prototypes (J, H, P, M, respectively) presented as images and mock-ups in six focus groups in Peru and in Thailand for discussion and ranking.

**Fig 4:**
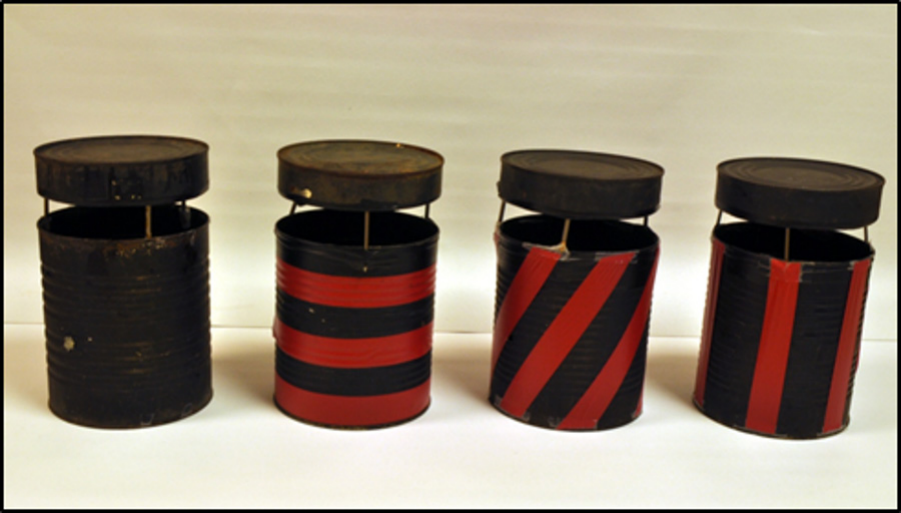
Evaluation of cans with stripe patterns compared to black cans. Stripe patterns on black cans evaluated in walk-in cage, sticky panel bioassays. Cans were evaluated at the same time.

The focus of phase 4 was on incorporating the preferred features into the design and testing of 4 mock-up prototypes (Fig 3) to assess trap attractiveness to *Aedes* and killing efficacy inside walk-in bioassay rooms (4 m wide x 4 m long x 2 m high). In addition to testing these four trap models in people’s homes for community acceptability of each of the designs, efficacy testing was being carried out. A 3.8 L (= one gallon) tin can that was painted flat black on the inner and outer surfaces was used as a “gold standard” control oviposition container, to which all other trap designs were compared. The responses of gravid mosquitoes to the black can configured with different patterns of red stripes was evaluated in a walk-in sized bioassay room using a sticky-panel bioassay. The black tin cans were modified by adding diagonal, horizontal and vertical red stripes using electrical tape (Fig 4) to determine if adding contrasting color patterns would increase the attraction of gravid mosquitoes to black cans. Tops were suspended above each can with nails fastened to the inside with glue. Responses to the stripe patterns were evaluated in a 4-choice assay by placing one striped can or a solid black can in each corner of the bioassay room or 1 meter apart in the middle of the room. The experiments were repeated 4 times, rotating the positions of the cans each time. Cans were filled with 500 mL well water and black sticky panels were inserted into each can; no chemical attractants were added. For each trial, 50 gravid New Orleans (NOLA) strain *Ae*. *aegypti* mosquitoes were released into each walk-in room. After a 24-hour exposure, each trial was terminated and cans were retrieved and the number of females trapped on each sticky panel was counted.

**Fig 5:**
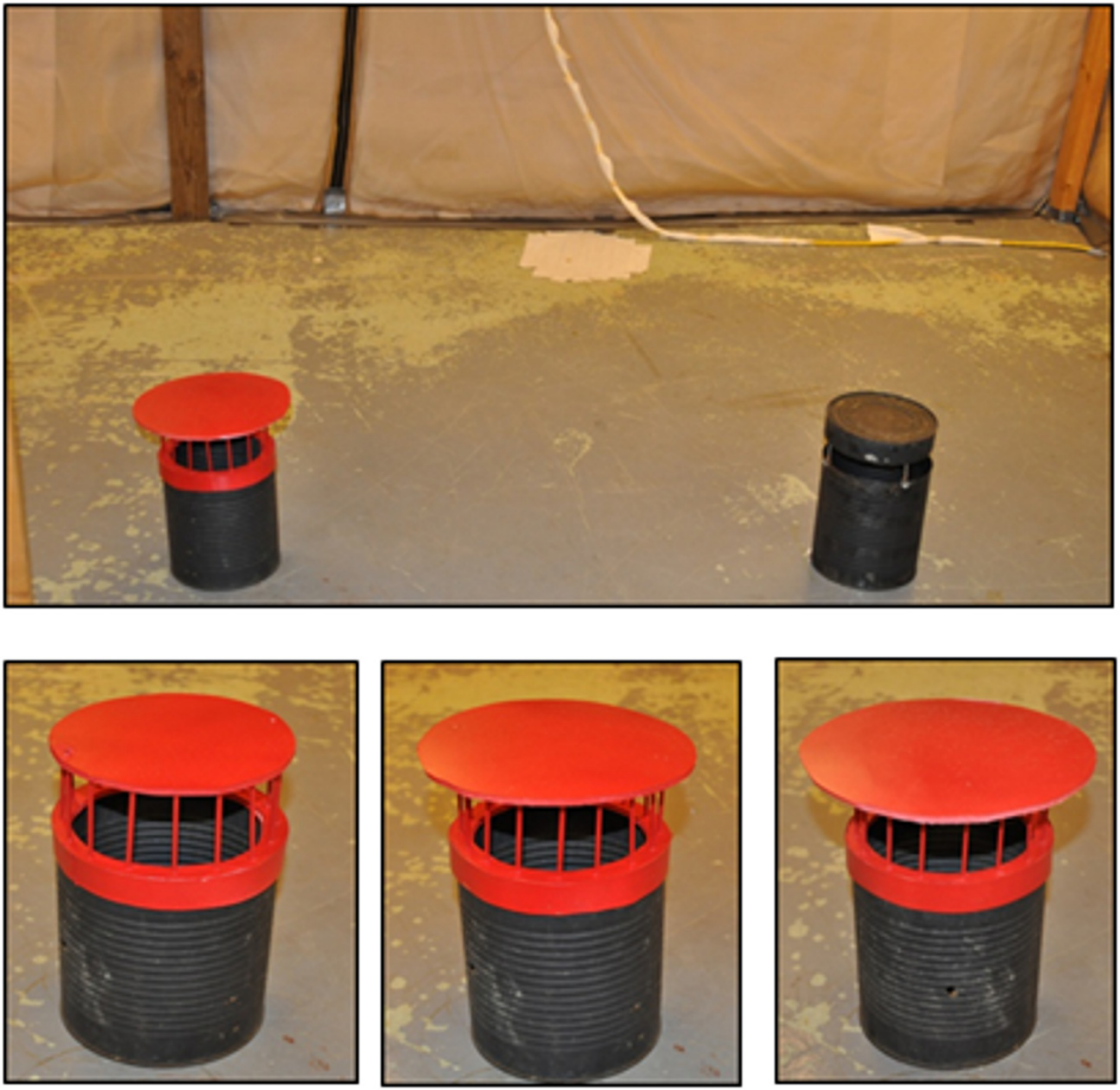
Depiction of evaluation of various trap prototypes. Top panel: Walk-in bioassay room showing placement of red cover and black cover cans. Bottom panel: Black cans fitted with small, medium and large sized covers (left to right).

Next, red colored covers were added to the black cans (Fig 5), which were also tested in walk-in rooms using the sticky-panel bioassay. Effects of color and size of the cover of the trap were investigated using 3 different sized covers (Fig 5). We used two-choice sticky panel bioassays to compare the performance of a black one-gallon can with a black cover to a black can with a red cover. We evaluated each of the three cover sizes four times. Well water (500 mL) was added to each can, which also contained a sticky panel, and 50 gravid NOLA strain *Ae*. *aegypti* mosquitoes were released for each assay.

Finally, solid red and solid black traps were tested under high and low light conditions to evaluate performance at different light intensities. Effects of light level on the responses of gravid NOLA strain *Ae*. *aegypti* to a red and black one gallon tin cans fitted with covers were evaluated in walk-in bioassay rooms. Red cans were constructed by wrapping red velour paper around black cans. Two identical cans were placed in diagonally opposite corners of the room. One corner was dimly lit (1.5 lux) while the opposing corner had 25X brighter light levels (38 lux). Each trial consisted of rotating the cans through all four corners of the room. Four trials were completed resulting in 16 replicate evaluations. The cans were filled with 500 mL well water and each was fitted with a sticky screen. Fifty gravid NOLA strain *Ae*. *aegypti* were released into each of three walk-in rooms on the same day. After a 24-h exposure period, the numbers of females caught on each sticky screen were counted. In all 2-choice and 4-choice bioassays we recorded the percentage of females that were trapped in each experimental trap relative to the total number of females trapped in the other traps used in the same bioassay.

Phase 5 activities included FGD and testing of traps inside homes. A total of 4 FGD (n = 48 total participants) in Iquitos and 4 FGD in Lopburi (n = 40 total participants) were conducted to discuss the four trap design options, which were presented to them physically as mock-ups. As before, individuals were asked to comment on features they liked and disliked, recommendations for changes, and perceived challenges related to the use of traps. A second activity focused on testing the 4 traps inside homes selected using convenience sampling. Two traps (the 1^st^ choice of traps from FGD in Peru and Thailand) were placed in each of four homes, and two different traps (the 2^nd^ choice from FGD in both Peru and Thailand) were placed in each of four different homes. All traps were used for one week inside the selected house. Feedback about the traps was obtained via semi-structured interviews from participants at day 3 and at day 7. This feedback was then presented to the design and entomology teams for additional walk-in room testing, and design and construction of the final trap model.

**Fig 6:**
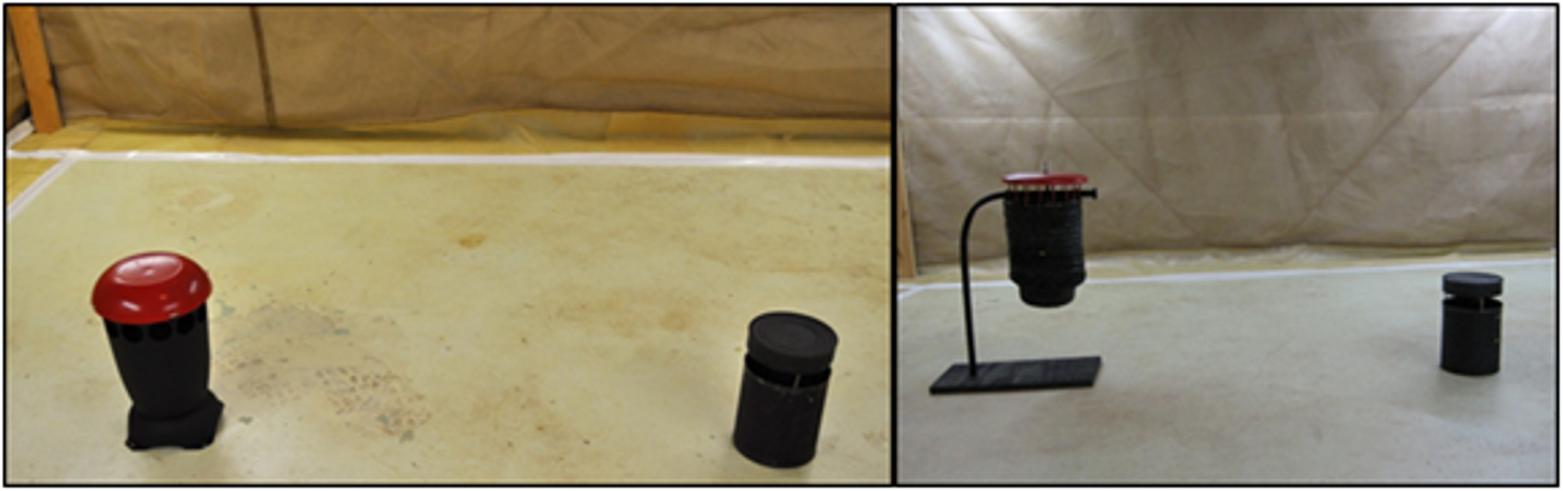
Depiction of evaluation of efficacy of two trap models. H (oval) and J (lantern) traps compared to black tin can with cover in walk-in bioassay room.

Two trap designs were evaluated in comparison to the standard black tin can. Trap “H” which was oval in shape with a red cover and the “J” lantern had an accordion hanging design (Fig 6). Design “H” also included netting shaped into a funnel which was expected to further trap mosquitoes along with the sticky screen. We used a two-choice, walk-in room, sticky screen bioassay to compare the responses of gravid NOLA strain *Ae*. *aegypti* to the “H” trap in the presence of the standard black one gallon can. Well water (300 or 500 mL) was added to each black can and either 300 or 500 mL well water were added to the “H” trap. Gravid NOLA strain *Ae*. *aegypti* (n = 50) were released into each assay. The last phase focused on modifying a near-final prototype. This prototype was then presented to the research team and, in Peru, where the trap would be evaluated first, to vector control officials to obtain feedback before mass production of the final version of the trap.

**Fig 7:**
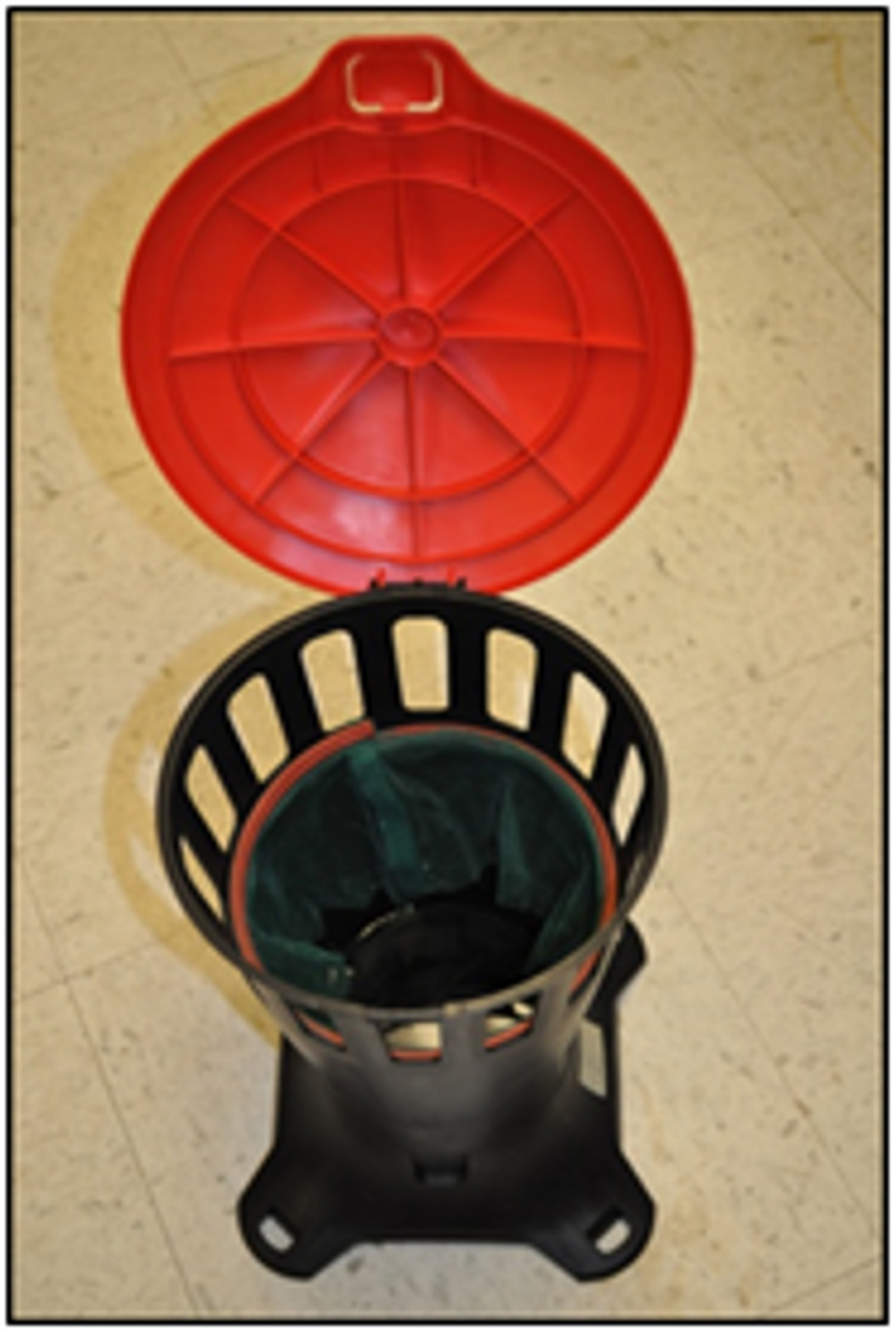
Depiction of Model P prototype. Model P prototype trap showing Duranet® netting.

The model P trap was evaluated against gravid Iquitos strain *Ae*. *aegypti* (n = 50) in walk-in room bioassays. The trap lined with green Duranet® netting (Fig 7) and filled with 500 mL of Raleigh (North Carolina) tap water was placed in the center of the room. Tap water, rather than well water, was used to simulate conditions of trap use by homeowners. In previous research [26–27] we showed that plant infusions contained semiochemicals that attracted and stimulated gravid *Ae*. *aegypti* to oviposit. We optimized the activity of plant infusion over a range of plant biomass and fermentation times [28], isolated bacteria from the infusion and showed that the bacteria were the source of the attractants for gravid *Aedes* mosquitoes [29]. Subsequently, a mixture of the four most bio-active bacteria—Isolate B1 (Bacillus thuringiensis), B5 (Lactococcus lactis), B13 (Citrobacter freundii), and B14 (Brevundimonas vesicularis)—were encapsulated with calcium alginate lyophilized and vacuum-sealed with a larvicide, spinosad, to produce an Attract and Kill (A&K) pouch for long term storage. Each A&K pouch contained 80 mg spinosad (Natular™ XRT) granules and 100 mg lyophilized bacterial beads. When traps were set up, the contents of an A&K pouch were added to the trap, which contained 500 mL of water. The age of the bacterial beads from lyophilization to deployment ranged from 56 to 81 days.

## Results

**Fig 8:**
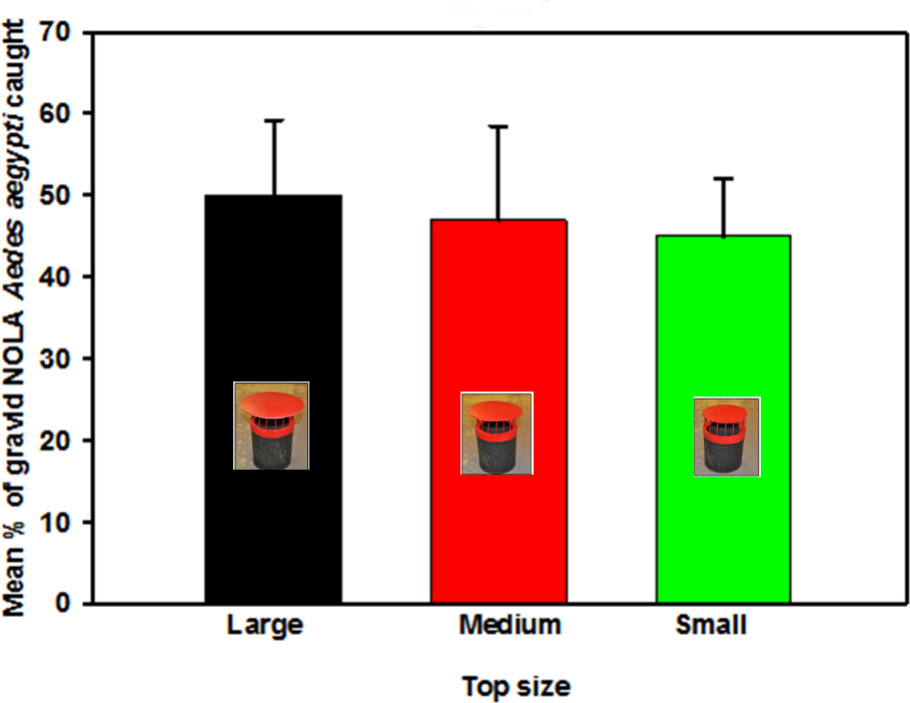
Evaluation of red trap covers of different sizes. Mean percentage (± SD) of gravid NOLA strain *Aedes aegypti* caught on sticky panels in black cans fitted with different sized red covers. Each trap with a red cover was tested against a black one gallon tin can fitted with a black cover. Traps were placed 1 m apart in the center of a walk-in cage (n = 4).

**Fig 9:**
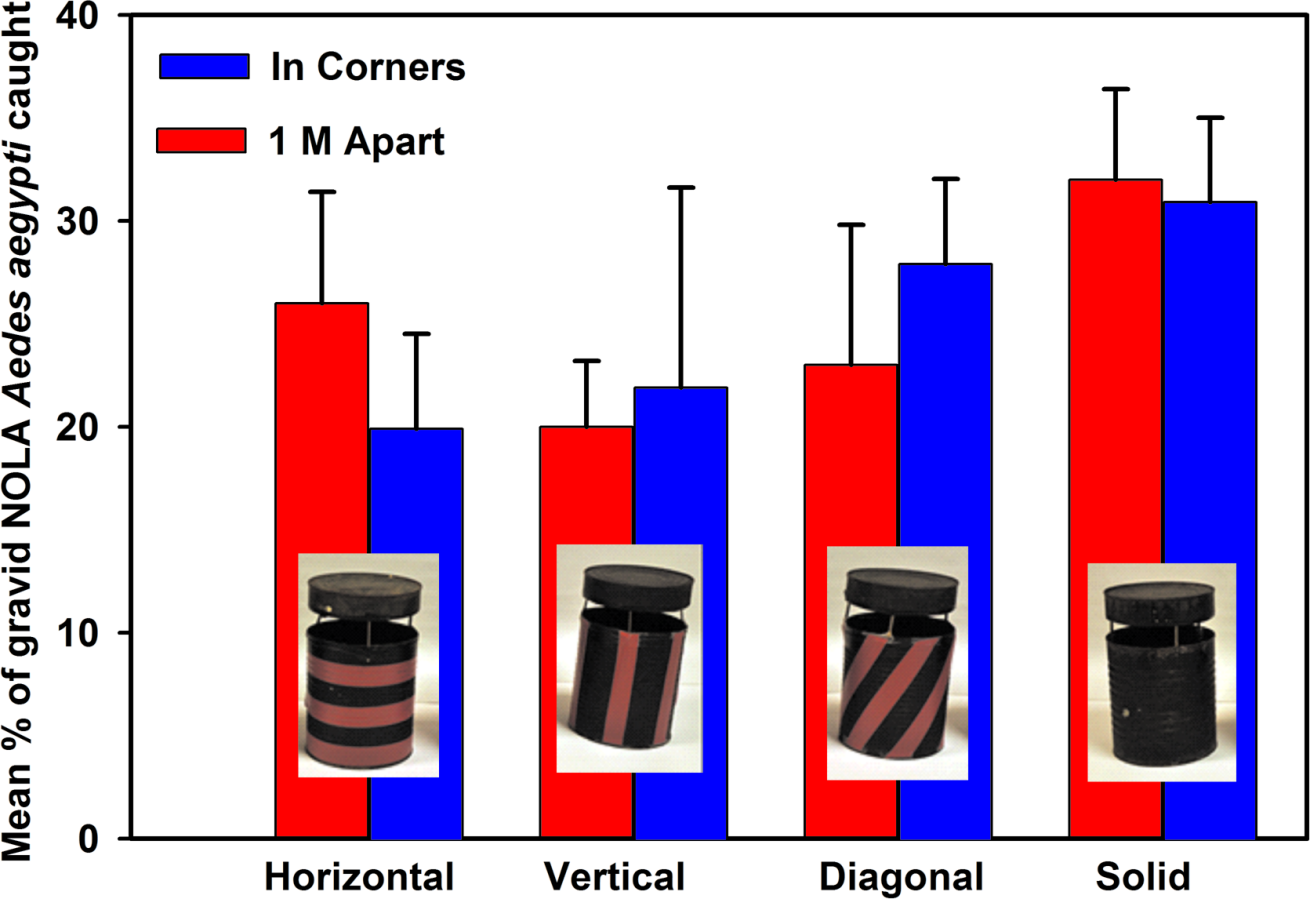
Evaluation of trap prototype based on room placement in the walk-in room. Mean percentage (± SD) of gravid NOLA strain *Aedes aegypti* caught on sticky panels in red striped cans with black tops placed 1 m apart in the center or in corners of a walk-in room (n = 4). Percentages represent females that were trapped in the experimental trap relative to the total number of females trapped in all four traps used in each assay, i.e., the sum of all percentages for each experimental design equals 100%.

Preferences regarding characteristics of the trap emerged in the first five focus group discussions (S1 Table in supplemental information). Community members wanted dark traps because “*dark colors attract mosquitoes*”, though efficacy studies indicated that the addition of a red cover did not reduce attractiveness to mosquitoes. FGD participants also described ideal traps being about 30 cm tall, and made of durable materials that would not create more trash (vs. recyclable parts, even if biodegradable). They were also concerned about the trap safety for young children and pets, with much of the discussion focused on ways to ensure that no one accidentally ingested the larvicide inside, and this discussion included the possibility of secure covers or putting traps within something like a “*chicken cage*”. In 2-choice walk-in room bioassays, we recorded the percentage of females that were trapped in the experimental trap relative to the total number of females trapped in both traps. Trap cover size affected mosquito response to the traps, with traps with larger covers trapping more females (Fig 8). Black cans fitted with small and medium red covers caught slightly, but insignificantly, fewer females than control traps with black tops (Fig 8). Traps fitted with the largest red cover caught as many females as the control black top traps. In addition, varying red patterns on a black trap body failed to increase trapping efficacy in 4-choice assays (Fig 9).

**Fig 10:**
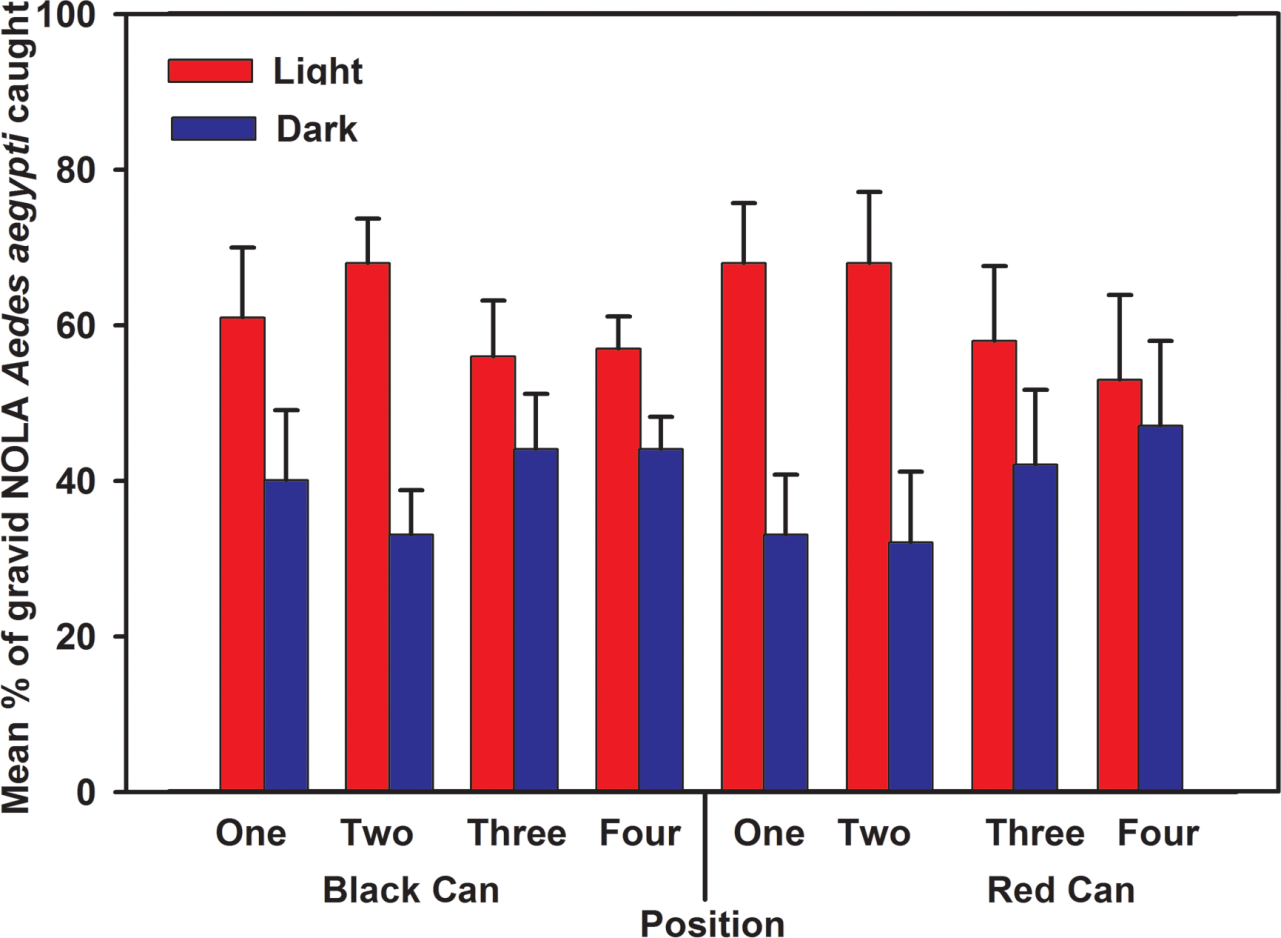
Evaluation of trap prototype based on exposure to different light levels in walk-in bioassay cage. Mean percentage (± SD) of gravid NOLA strain *Ae*. *aegypti* caught on sticky screens in red and black cans placed in diagonal corners of walk-in bioassay cages. One corner was dimly lit while the opposite corner had normal light levels.

Participants also specified locations they might choose to place traps in their homes, such as bathrooms because “*mosquitoes are attracted to humid places like bathrooms and kitchens*”. However, in the lab, lighting was found to be important, which could influence placement location. Sticky panel tests in varied light levels indicated that trap catch was highest when traps were positioned under brighter light, regardless of trap design (Fig 10). The traps were rotated through the four corners of the walk-in assay room with alternate lighting in a factorial manner to control for possible position effects.

The discussion related to the photographs at both study sites focused on home features to consider during trap design, such as long narrow homes combined with dirt floors that might make the traps vulnerable to being toppled over by individuals walking through. The design team initially considered whether communities might prefer modern or chic designs, or traps made of innovative materials; after FGD and viewing photographs of both study areas, it was understood that, for purposes of acceptability, other trap features, such as stability and durability of materials, were more important to the communities.

The presentation of 22 trap design options during the video conference call (phase 2) between the design and research teams produced a discussion of aesthetics, features, and requirements that must be met to maintain efficacy during use. Notable points related to aesthetics, ecological impact, trap stability and installation procedures (S2 Table in supplemental information for more details). For example, researchers noted that some traps were more visually appealing than others; but it was also noted that one design looked like a “*collapsible*, *can-shaped lantern*”, which could be altered in the community to be used as such. The refill chamber for some designs consisted of a bowl with a paper lid, which could drastically increase debris in the environment. The ease with which the larvicide, attractants and water could be refilled by individuals in the community was also a main consideration of residents. Lastly, residents wanted the trap to be easy to install and “*stable enough*” to be used outdoors–something that the community had valued in the first series of FGD, and important to consider because some of the study areas were prone to flooding–even indoors–with strong rains.

The design team narrowed down trap design options to six that combined features that were discussed in the conference call and initial FGDs (Fig 2). During the 9 FGD at each study site (Iquitos, n = 81 participants; Lopburi, n = 90 participants), each of these images was presented individually to the participants, discussed extensively, and the trap design options were ranked. The FGD participants’ first comments after seeing each trap primarily focused on functional analogies (*i*.*e*. concept design "G" “*looked like a bucket*”, "I" like “*a blender*” and "J" like “*a lantern*”) (S3 Table) vs. how well each might work.

Certain behavioral challenges associated with using each trap were identified by the questions that were being asked as well. For example, based on the questions, it was evident that some people were concerned about the inconvenience of refilling the trap and wanted to increase the time before refilling was required, or they might consider re-designing a trap to insert a light bulb. People also inquired about making their own traps with similar materials. When asked to rank the traps, the same top two choices emerged at both study sites: one which looked like “*a Chinese lantern*” (model "J") because “*it’s more aesthetic*” and “*it could be decorative*”, and because it could be secured to a wall, which was deemed safer for children. However, participants in Lopburi expressed concerns about model J’s durability. The second choice was model "H" because its rain-release holes were practical, it had a bigger cover so it could be placed in the backyard, and the netting material was the only part to replace. Trap “H” was also found to have the consistently highest catch of gravid females in walk-in room trials.

Based on feedback to date, the design team worked on narrowing choices to four designs to create mock-ups for presentation to the community and further entomological testing (Fig 3). Four additional FGD were conducted with community members on the 4 traps at both sites (S4 Table summarizes the discussion).

Additional data were collected from semi-structured interviews with residents of households where traps were placed for pilot testing and observation; the top two trap choices were placed in 4 houses each (*i*.*e*. two model “J” traps per house in four houses, two model “H” traps per house in four houses), and feedback about the traps was obtained from participants at day 3, and at day 7. For the most part, as requested, most residents did not interact with the traps and did not have much to say about the traps. Most noticed that the liquid in the trap became slightly dirty over the course of the week (dust, one or two different types of insects), and a few mentioned that *“we know the trap works because we saw fewer mosquitoes in our house*.*”* When asked if they would move the trap or if it had been inconvenienced daily activities, none said it was inconvenient, but a few commented that they might move it to a location where they have seen more mosquitoes. One difficulty experienced with the model “J” was that the ducks in the house of one of the participants pecked away at the soft accordion-like sides of the trap (this was a mock up, so even though it was possible that the final version would be made of more durable materials, it was clear that it did not hold up as well as the other trap model).

**Fig 11:**
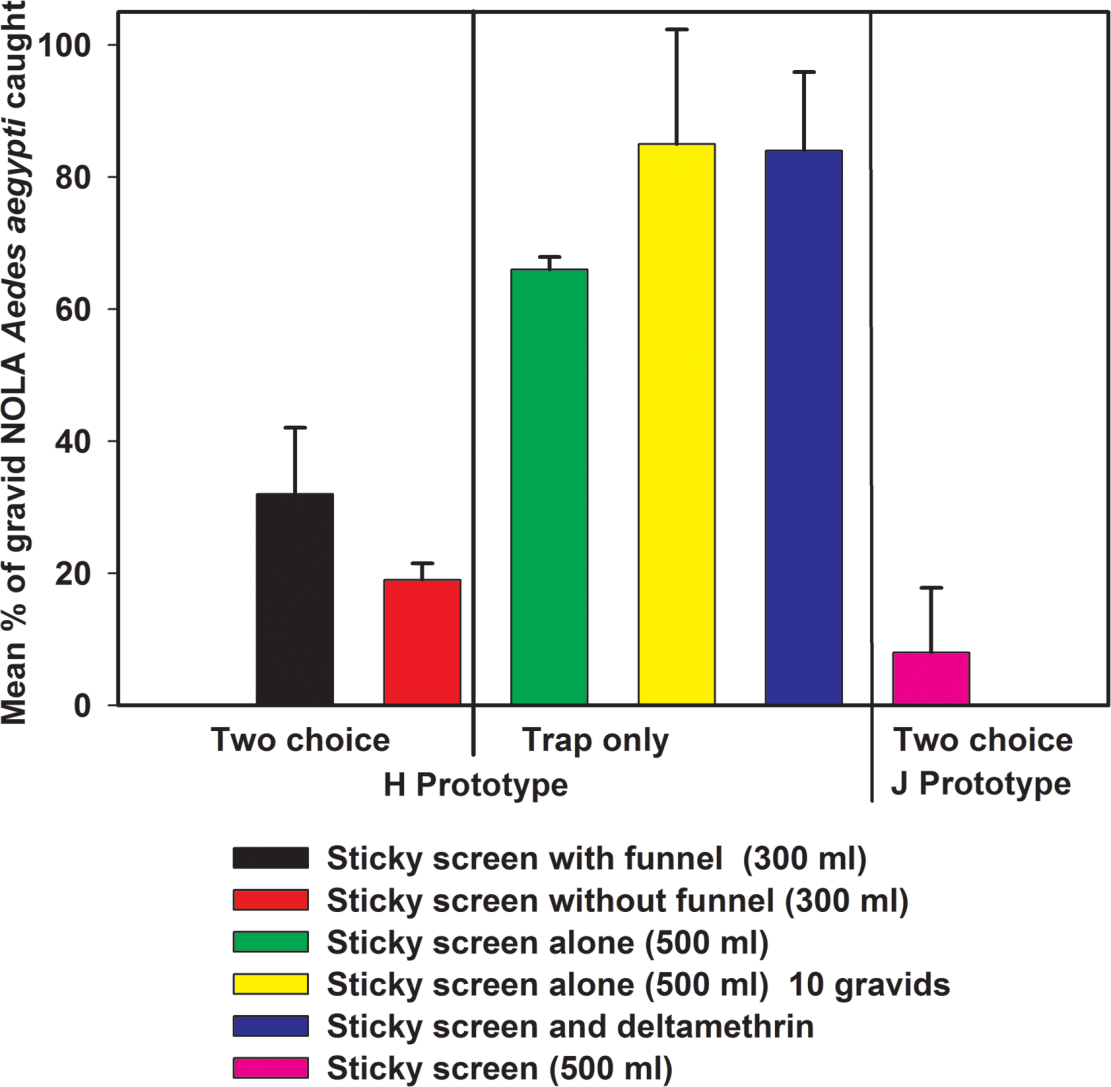
Evaluation of different prototypes with and without funnels, and with different volumes of well water. Mean percentage (± SD) of gravid NOLA strain *Ae*. *aegypti* caught on sticky screens in HLB “H” and “J” traps in walk-in cage bioassays (n = 4). Some assays were the two-choice and included a one gallon black can (n = 4).

In the lab, two trap designs were evaluated in comparison to the standard black, one gallon tin can: model “H” which was oval in shape with a red cover and the lantern-looking model “J” with an accordion hanging design. All three traps were fitted with sticky screens and the numbers of mosquitoes caught 24 hours after being released were compared for all three traps. The lantern model “J” was difficult to set up with the sticky screen, and similar to the “H” trap, performed poorly compared to the black tin can fitted with a cover. We also evaluated the sticky screen in the “H” trap with and without the funnel and found that the funnel potentially impeded the entrance of females into the trap (Fig 11). Reducing the density of mosquitoes from 50 to 10 females increased the mean percent of mosquitoes caught by trap “H”. Furthermore, reducing the volume of well water from 500 to 300 mL greatly reduced the trap catch on the sticky screens (Fig 11).

**Fig 12:**
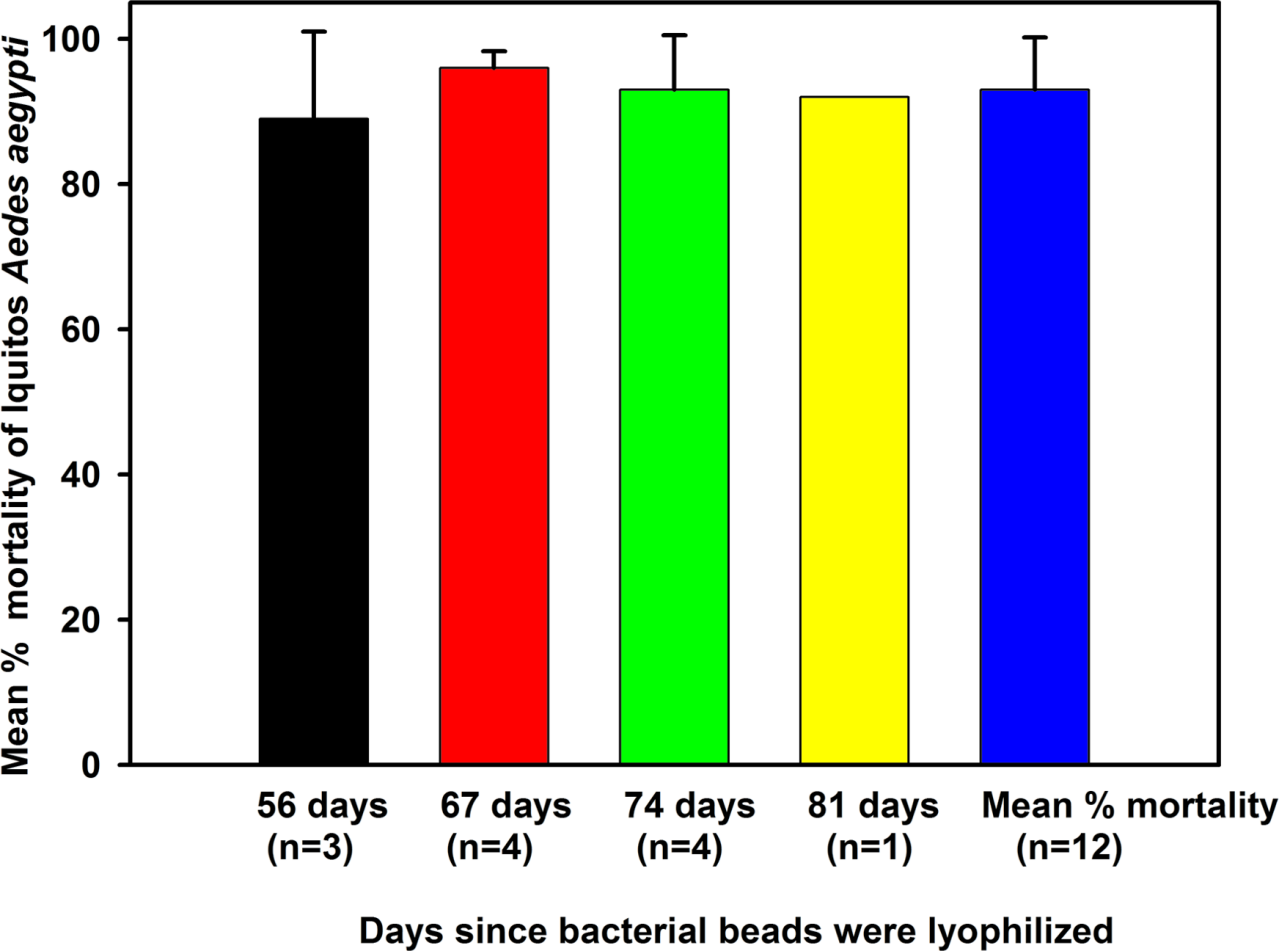
Evaluation of gravid Iquitos *Ae*. *aegypti* mortality based on bacterial bead lyophilization date. Mean percentage (± SD) mortality of gravid Iquitos strain *Ae*. *aegypti* (n = 50) exposed for 24-h to HLB second “H” trap prototype in walk-in bioassay cages. Each trap contained 500 mL of Raleigh tap water and the contents of one A&K pouch (80 mg spinosad and 100 mg bacterial beads). Dates correspond to the dates that bacterial beads were lyophilized.

The model P prototype (Fig 7) was evaluated against gravid Iquitos strain *Ae*. *aegypti* in walk-in cage bioassays comparing different lots of lyophilized bacterial beads aged in the room temperature. Average percent mortality was high for bacterial bead pouches aged for 56 to 81 days (Fig 12).

**Fig 13:**
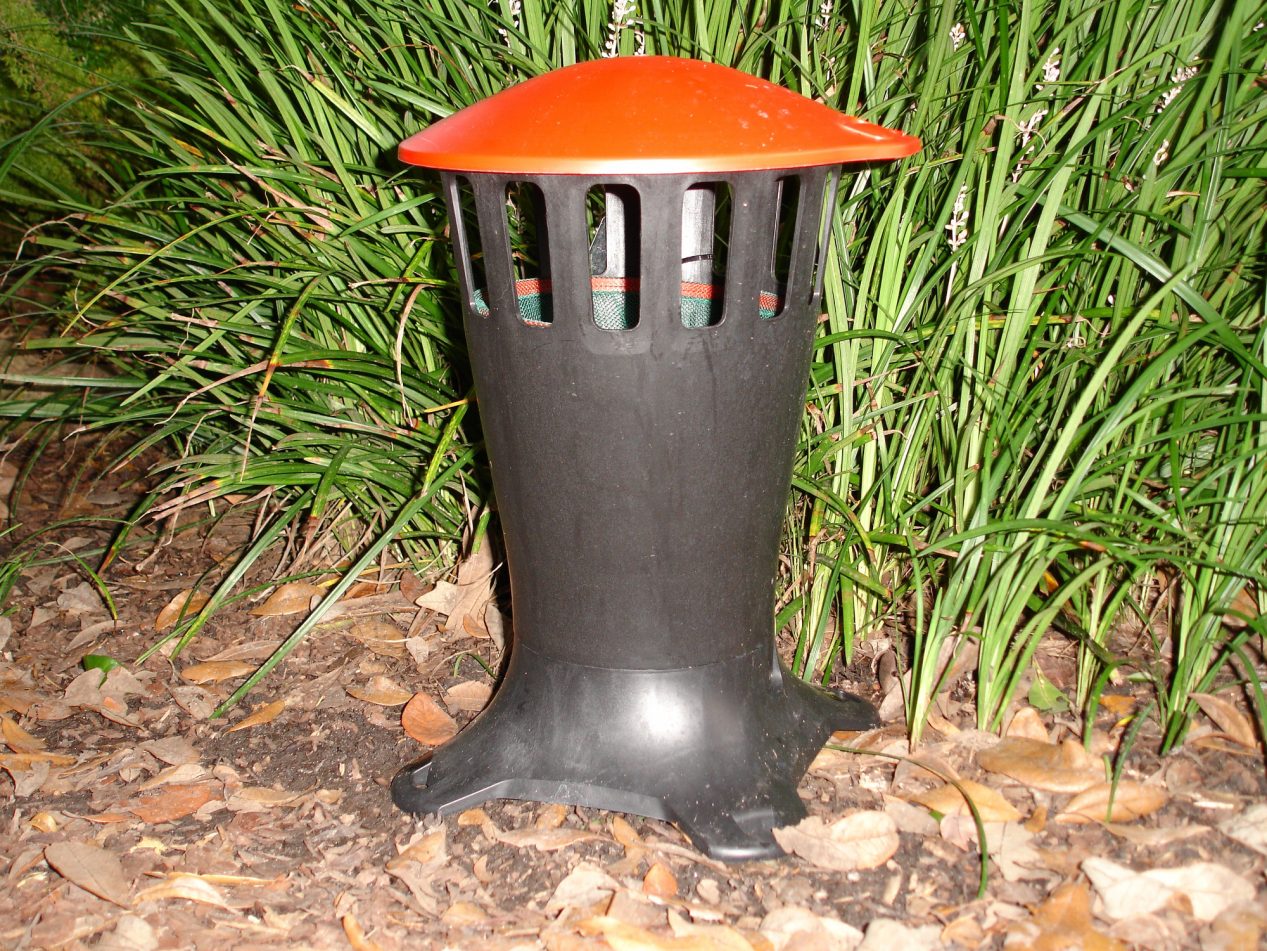
Design of final prototype.

Results from both FGD and pilot testing in houses suggested trap model “P” would be most accepted and durable. Slight improvements were made to model “P” based on the feedback from the community and our research team, and this model was presented to vector control officials in a focus group format (Fig 13). Vector control officials reiterated issues related to rain cover (*i*.*e*. that the lid be large enough to prevent rain from entering) and stability of the trap. It was also noted that the internal net in the trap would last longer if it was not immersed in the liquid, and they were also very interested in understanding how the trap components all worked together. The sticky panel tests with mock traps tested with various cover sizes indicated that the highest response of gravid female mosquitoes was to the mock trap with the largest cover size.

Finally, FGD participants were asked how much they thought they would pay for these traps and their components. Their responses ranged from US $8.00–20.00 for the actual trap (most said US $8.00, but one said US $20.00) in Peru, and US $3.00–5.00 (but not higher than US $10.00) in Thailand. In both countries, participants mentioned that some traps–with more durable parts–might be slightly more expensive than others. In Peru, participants also gave us a range of the various parts: US $2.00–7.00 for the net in the trap interior, and US $0.30–2.00 for the beads/larvicide.

## Discussion

This study presents findings from a participatory research project in which the ALOT trap was developed with features relevant for the individual resident and communities where they were to be later be evaluated. There are several key findings from this study. First, while the process presented herein does not capture population based data on the acceptability and use of the traps (note: results on whether this participatory approach yielded a product that is used by the community will be presented in a separate paper), it does provide a starting point for discussions on how to increase use, which is consistent with processes developed for other disease control interventions [30–32]. The participatory process described herein ensured that the living situations and realities of the community that would be using the traps were always in the forefront of the discussions. For those involved in the process, there was a clear iterative presentation of options, consideration of different perspectives, and interdisciplinary exchange that allowed the development of a novel trap–and yet this process was always grounded in the efficacy testing that was occurring simultaneously in the lab, and the feedback received from the community FGDs regarding preferences for their home use.

Second, there are numerous examples of public health interventions that demonstrated efficacy at preventing and reducing morbidity and mortality associated with particular diseases (i.e., vaccines, bednets, condoms), but for which adoption processes varied in time and amount [33–40]. Community acceptability is key for adoption; working with the community in the design process is important for multiple reasons. A similar intervention for combatting a vector-borne disease is the bednet for malaria prevention. One of the lessons from the bednet experience was that effectiveness of the bednets did not depend solely on their availability, as initially there were many programs that gave nets out for free, yet people did not always use them. A second early lesson of the bednet development process related to wide range of use as a function of color, level of privacy, and texture, in addition to cost [41–42]. Future manuscripts will focus on the trap effectiveness within the community and long-term acceptability of these traps, but there was a high initial acceptance of trap placement in homes in Iquitos, Peru and in Lopburi, Thailand (though it was initially planned, the proof of concept portion of the study was not carried out in Thailand)—and we believe it was due to the participatory process that took place in the trap development.

Lastly, despite different landscape, culture, language, and types of homes, the discussions with community members in Peru and Thailand were surprisingly similar and, thus, present unique lessons learned from this study that may be relevant for other vector-borne disease control tool development processes. All FGD were very structured, with a tool to ensure that similar topics were discussed with each trap. However, ultimately, several common themes appeared to guide trap preference in both communities: safety of traps to young children in households, durability in rainy and windy weather, cost, environmental impact, the ease of trap maintenance (including refilling). Aesthetics were secondary in both settings; but again, surprisingly, trap rankings made in both settings were very similar, with the same top choice for a trap. In hindsight, it was key to use a very structured and directed focus group guide since it allowed for an effective comparison of findings from both locations. However, we believe that we could have obtained similar feedback from the community with fewer focus groups (there were a total of 32 FGD).

The importance of involving the community in the trap development process is evident in multiple ways. When the US-based design team initially showed trap designs and concepts, much of their excitement focused on novel materials, shapes and textures that could be used–this excitement was short-lived, as local social scientists who knew the communities well also knew that traps that were too chic or novel might be subject to unwanted attention and possible theft. Traps needed to be attractive enough for people to have in their homes, but not desirable objects for theft. Several discussions focused on the possibility of using the type of items that people do not steal–such as recyclable plastic bottles, as these materials would likely not be considered valuable given the ubiquitous nature of these materials.

The entomology team was also able to integrate findings from the community in the large room bioassays. Initially, we thought the traps would be black, but results from the Thailand focus groups led us to reconsider the possibility of testing other colors. The bioassays revealed that there seemed to be no differentiation between black and red to mosquitoes based on gravid mosquito response to sticky panel bioassay testing. These results are consistent with poor sensitivity to wavelengths in the red region of the color spectrum in most mosquito species [43]. As a result, and after various modifications to the design and cage trials, the final trap was mostly black with a red lid.

Through this process, we also identified relevant but unexpected issues, such as potential misuse of the trap: the use of a trap as a lamp, the possibility that people would take traps with them to rice fields or move them around the house. Our participatory approach also highlighted the value of integrating the perspectives of interdisciplinary research and product development groups with community preferences to develop a useful product that is relevant to the community. Our next challenges include designing an approach to evaluate the effectiveness and use of this product at the population level for reducing both dengue vectors and dengue infection.

### Limitations

There are several limitations worth noting. First, results based on convenience sampling may not represent the full spectrum of opinions of the general community; the; the use of FGD to generate data on design preferences also produces information that may not be truly population-based, nor do FGD necessarily reflect broad community participation in the process. Population-based surveys and observational studies of traps being piloted in the field will be necessary to assess trap acceptability, use and long term adoption. Second, during the final stages (Phase 5) of presenting mock-ups of the trap designs to FGD participants and to households that piloted them in Iquitos and Lopburi, we did not present a final, polished product that may have influenced their trap preferences. We presented them with the hand-sketched images of the four models, and a mock-up that would allow them to get a better feeling of what this trap would look like assembled–and in the case of the four households that piloted each model, they received two mock ups per house. But the mock-up materials were not as durable as the final products: in one mock-up model, a few of the connectors between the trap and lid broke. These experiences and observations with the mock ups likely influenced their responses to each model. A third limitation relates to input only from Peru and Thailand. Given that there are likely differences in cultural and environmental characteristics, the trap may be better suited in one environment over the other, or at different times of the year. Given that logistical considerations necessitated the design of only one prototype for testing, in lieu of multiple prototypes at each site, it is difficult to assess which design is truly better suited for a specific environment. Lastly, input from government vector control experts was only acquired towards the end of the design process. Important cultural and programmatic factors considered earlier in the process could have influenced the design.

## Conclusions

Iterative discussions between an interdisciplinary team comprised of entomologists, social scientists, industrial designers, and the targeted community facilitated the development of an oviposition trap for *Aedes* container inhabiting species with characteristics and components that were entomologically sound and acceptable to community members. Beyond the development of a trap with acceptable characteristics based on safety, durability, maintenance and aesthetics, discussions also alerted the research team to potential misuse or improper use of the traps that could have hindered evaluation studies on vector control and dengue prevention.

## Supporting Information

S1 Table Summary of results from first 5 focus group discussions in Iquitos (Phase 1). (DOCX)

S2 Table Summary of discussion points between research and design teams about initial 22 trap designs regarding features needed and wanted for the traps (Phase 2). (DOCX)

S3 Table Summary of nine focus group discussions regarding 6 trap models in Peru and Thailand (Phase 3). (DOCX)

S4 Table Summary of four focus group discussions with community members in Iquitos and Lopburi discussing four trap models (Phase 5). (DOCX)

## References

[1] Gubler DJ. The global emergence/resurgence of arboviral diseases as public health problems. Arch Med Res. 2002 Jul-Aug;33(4):330–42. . 12234522 10.1016/s0188-4409(02)00378-8

[2] Bhatt S, Gething PW, Brady OJ, Messina JP, Farlow AW, Moyes CL, et al The global distribution and burden of dengue. Nature. 2013 4 25;496(7446):504–7. 10.1038/nature12060 23563266 PMC3651993

[3] Weaver SC, Forrester NL. Chikungunya: Evolutionary history and recent epidemic spread. Antiviral Res. 2015 8;120:32–9. 10.1016/j.antiviral.2015.04.016 . 25979669

[4] Dyer O. Zika virus spreads across Americas as concerns mount over birth defects. BMJ. 2015 12 23;351:h6983 10.1136/bmj.h6983 . 26698165

[5] Scott TW, Morrison AC, Lorenz LH, Clark GG, Strickman D, Kittayapong P, et al Longitudinal studies of Aedes aegypti (Diptera: Culicidae) in Thailand and Puerto Rico: population dynamics. J Med Entomol. 2000 1;37(1):77–88. . 15218910 10.1603/0022-2585-37.1.77

[6] Harrington LC, Edman JD, Scott TW. Why do female Aedes aegypti (Diptera: Culicidae) feed preferentially and frequently on human blood? J Med Entomol. 2001 5;38(3):411–22. . 11372967 10.1603/0022-2585-38.3.411

[7] Sikka V, Chattu VK, Popli RK, Galwankar SC, Kelkar D, Sawicki SG, et al The emergence of Zika virus as a global health security threat: a review and a consensus statement of the INDUSEM Joint Working Group (JWG). J Glob Infect Dis. 2016 Jan-Mar;8(1):3–15. 10.4103/0974-777X.176140 27013839 PMC4785754

[8] Ndeffo-Mbah ML, Durham DP, Skrip LA, Nsoesie EO, Brownstein JS, Fish D, et al Evaluating the effectiveness of localized control strategies to curtail Chikungunya. Sci Rep. 2016 4 5;6:23997 10.1038/srep23997 27045523 PMC4820747

[9] Achee NL, Gould F, Perkins TA, Reiner RC Jr, Morrison AC, Ritchie SA, et al A critical assessment of vector control for dengue prevention. PLoS Negl Trop Dis. 2015 5 7;9(5):e0003655 10.1371/journal.pntd.0003655 25951103 PMC4423954

[10] Morrison AC, Zielinski-Gutierrez E, Scott TW, Rosenberg R. Defining challenges and proposing solutions for control of the virus vector Aedes aegypti. PLoS medicine. 2008;5(3):e68 10.1371/journal.pmed.0050068 18351798 PMC2267811

[11] Reiter P. Surveillance and control of urban dengue vectors. In: Gubler DJ, Ooi EE, Vasudevan S, Farrar J, C.A.B. Dengue and dengue hemorrhagic fever. 2nd ed. Boston, MA; 2014. p. 484–521.

[12] Eisen L, Beaty BJ, Morrison AC, Scott TW. Proactive vector control strategies and improved monitoring and evaluation practices for dengue prevention. J Med Entomol. 2009 11;46(6):1245–55. . 19960667 10.1603/033.046.0601

[13] Lorenzi OD, Major C, Acevedo V, Perez-Padilla J, Rivera A, Biggerstaff BJ, et al Reduced incidence of Chikungunya virus infection in communities with ongoing *Aedes aegypti* mosquito trap intervention studies—Salinas and Guayama, Puerto Rico, November 2015-February 2016. MMWR Morb Mortal Wkly Rep. 2016 5 13;65(18):479–80. 10.15585/mmwr.mm6518e3 27171600

[14] Wesson DM, Morrison AC, Paz Soldan VA, Moudy RM, Long K, Ponnusamy L, et al Update on evaluation of an attractive lethal ovitrap (ALOT) against Aedes aegypti for dengue control in Iquitos, Peru. Am J Trop Med Hyg. 2013;89(5) Suppl 158 [cited 2016 July 6]. Available from: http://www.astmh.org/ASTMH/media/Documents/2013AbstractBook501thru750.pdf

[15] Ponnusamy L, Schal C, Wesson DM, Arellano C, Apperson CS. Oviposition responses of *Aedes* mosquitoes to bacterial isolates from attractive bamboo infusions. Parasit Vectors. 2015 9 23;8:486 10.1186/s13071-015-1068-y ; Pubmed Central PMCID: PMC4581471. 26399712 PMC4581471

[16] Instituto Nacional de Estadística e Informática, Perú [Internet]. Estimaciones y proyecciones de población total por sexo de las principales ciudades, 2000–2015. 2009 Feb 17;(23) [cited 2015 Feb 17]. Available from: http://proyectos.inei.gob.pe/web/biblioineipub/bancopub/Est/Lib1020/Libro.pdf

[17] Forshey BM, Reiner RC, Olkowski S, Morrison AC, Espinoza A, Long KC, et al Incomplete protection against dengue virus type 2 re-infection in Peru. PLoS Negl Trop Dis. 2016 2 5;10(2):e0004398 10.1371/journal.pntd.0004398 ; Pubmed Central PMCID: PMC4746126. 26848841 PMC4746126

[18] Forshey BM, Guevara C, Laguna-Torres VA, Cespedes M, Vargas J, Gianella A, et al Arboviral etiologies of acute febrile illnesses in Western South America. PLoS Negl Trop Dis. 2010 8 10;4(8):e787 10.1371/journal.pntd.0000787 ; Pubmed Central PMCID: PMC2919378. 20706628 PMC2919378

[19] Stoddard ST, Wearing HJ, Reiner RC Jr, Morrison AC, Astete H, Vilcarromero S, et al Long-term and seasonal dynamics of dengue in Iquitos, Peru. PLoS Negl Trop Dis. 2014 7 17;8(7):e3003 10.1371/journal.pntd.0003003 ; Pubmed Central PMCID: PMC4102451. 25033412 PMC4102451

[20] Morrison AC, Gray K, Getis A, Astete H, Sihuincha M, Focks D, et al Temporal and geographic patterns of Aedes aegypti (Diptera: Culicidae) production in Iquitos, Peru. J Med Entomol. 2004 11;41(6):1123–42. . 15605653 10.1603/0022-2585-41.6.1123

[21] Morrison AC, Minnick SL, Rocha C, Forshey BM, Stoddard ST, Getis A, et al Epidemiology of dengue virus in Iquitos, Peru 1999 to 2005: interepidemic and epidemic patterns of transmission. PLoS Negl Trop Dis. 2010 5 4;4(5):e670 10.1371/journal.pntd.0000670 20454609 PMC2864256

[22] National Statistical Office [Internet]. National Statistic Office of Thailand, Bangkok. The 2010 Population and Housing Census; 2010 [cited 2016 Apr 21]. Available from: http://web.nso.go.th/en/census/poph/cen_poph.htm

[23] Ministry of Public Health [Internet]. Department of Disease Control, Thailand; 2016 [cited 2016 Apr 21]. Available from: http://www.ddc.moph.go.th/eng/

[24] Apperson C, Czokajlo D, Kirsch P, Ayyash LA, Wesson D, Schal C. A novel lethal trap for gravid Aedes aegypti and Aedes albopictus. Am J Trop Med Hyg. 2007;77(5) Suppl 254.

[25] Bernard HR. Research methods in anthropology: qualitative and quantitative methods. 4^th^ ed. Lanham, MD: AltaMira Press; 2006.

[26] Ponnusamy L, Xu N, Nojima S, Wesson DM, Schal C, Apperson CS. Identification of bacteria and bacteria-associated chemical cues that mediate oviposition site preferences by Aedes aegypti. Proc Natl Acad Sci USA. 2008 7 8;105(27):9262–7. 10.1073/pnas.0802505105 ; Pubmed Central PMCID: PMC2443818. 18607006 PMC2443818

[27] Ponnusamy L, Wesson DM, Arellano C, Schal C, Apperson CS. Species composition of bacterial communities influences attraction of mosquitoes to experimental plant infusions. Microb Ecol. 2010 1;59 (1):158–73. 10.1007/s00248-009-9565-1 ; Pubmed Central PMCID: PMC4561554. 19641948 PMC4561554

[28] Ponnusamy L, Xu N, Böröczky K, Wesson DM, Abu Ayyash L, Schal C, et al Oviposition responses of the mosquitoes Aedes aegypti and Aedes albopictus to experimental plant infusions in laboratory bioassays. J Chem Ecol. 2010 7;36(7):709–19. 10.1007/s10886-010-9806-2 ; Pubmed Central PMCID: PMC4562425. 20521087 PMC4562425

[29] Ponnusamy L, Schal C, Wesson DM, Arellano C, Apperson CS. Oviposition responses of *Aedes* mosquitoes to bacterial isolates from attractive bamboo infusions. Parasit Vectors. 2015 9 23;8:486 10.1186/s13071-015-1068-y ; Pubmed Central PMCID: PMC4581471. 26399712 PMC4581471

[30] Maketa V, Vuna M, Baloji S, Lubanza S, Hendrickx D, Inocêncio da Luz RA, et al Perceptions of health, health care and community-oriented health interventions in poor urban communities of Kinshasa, Democratic Republic of Congo. PLoS One. 2013 12 19;8(12):e84314 10.1371/journal.pone.0084314 ; Pubmed Central PMCID: PMC3868617. 24367653 PMC3868617

[31] Lover AA, Sutton BA, Asy AJ, Wilder-Smith A. An exploratory study of treated-bed nets in Timor-Leste: patterns of intended and alternative usage. Malar J. 2011 7 21;10:199 10.1186/1475-2875-10-199 ; Pubmed Central PMCID: PMC3155971. 21777415 PMC3155971

[32] Gyapong M, Gyapong JO, Amankwa J, Asedem J, Sory E. Introducing insecticide impregnated bednets in an area of low bednet usage: an exploratory study in Northeast Ghana. Trop Med Int Health. 1996 6;1(3):328–33. . 8673835 10.1046/j.1365-3156.1996.d01-41.x

[33] World Health Organization. Updating on progress controlling yellow fever in Africa, 2004–2008. Wkly Epidemiol Rec. 2008 12 12;83(50):450–8. . 19069294

[34] Barnett ED. Yellow fever: epidemiology and prevention. Clin Infect Dis. 2007 3 15;44(6):850–6. . 17304460 10.1086/511869

[35] Lengeler C. Insecticide-treated bed nets and curtains for preventing malaria. Cochrane Database Syst Rev. 2004;2(2). 10.1002/14651858.CD000363.pub2 15106149

[36] Alonso PL, Lindsay SW, Armstrong Schellenberg JR, Keita K, Gomez P, Shenton FC, et al A malaria control trial using insecticide-treated bed nets and targeted chemoprophylaxis in a rural area of The Gambia, West Africa: The impact of the interventions on mortality and morbidity from malaria. Trans R Soc Trop Med Hyg. 1993 6;87 Suppl 2:37–44. . 8212109 10.1016/0035-9203(93)90174-o

[37] Aikins MK, Pickering H, Greenwood BM. Attitudes to malaria, traditional practices and bednets (mosquito nets) as vector control measures: a comparative study in five West African countries. J Trop Med Hyg. 1994 4;97(2):81–6. . 8170007

[38] D’Alessandro U. Insecticide treated bed nets to prevent malaria: The challenge lies in implementation. BMJ. 2001;322:249–50. 10.1136/bmj.322.7281.249. 11157510 PMC1119510

[39] Choi HW, Breman JG, Teutsch SM, Liu S, Hightower AW, Sexton JD. The effectiveness of insecticide-impregnated bed nets in reducing cases of malaria infection: a meta-analysis of published results. Am J Trop Med Hyg. 1995 5;52(5):377–82. . 7771600 10.4269/ajtmh.1995.52.377

[40] Wiseman V, Scott A, McElroy B, Conteh L, Stevens W. Determinants of bed net use in The Gambia: Implications for malaria control. Am J Trop Med Hyg. 2007;76(5):830–6. . 17488900

[41] MacCormack CP, Snow RW. Gambian cultural preferences in the use of insecticide-impregnated bed nets. J Trop Med Hyg. 1986 12;89(6):295–302. . 3806747

[42] Das ML, Singh SP, Vanlerberghe V, Rijal S, Rai M, Karki P, et al Population preference of net texture prior to bed net trial in Kala-Azar-endemic areas. PLoS Negl Trop Dis. 2007 12 12;1(3):e100 . 18160976 10.1371/journal.pntd.0000100 PMC2154387

[43] Allan SA. Physics of mosquito vision- an overview. J Am Mosq Control Assoc. 1994 6;10(Pt 2):2661–71. . 8965078

