# Supplementary material for: Design and Testing of Novel Lethal Ovitrap to Reduce Populations of Aedes Mosquitoes: Community-Based Participatory Research between Industry, Academia and Communities in Peru and Thailand
Source: PLoS One. 2016 Aug 17;11(8):e0160386. doi: 10.1371/journal.pone.0160386 (PMC4988764; doi:10.1371/journal.pone.0160386)
Supplement: S1 Table — (DOCX) [file pone.0160386.s001.docx]

**S1 Table**: Summary of results from first 5 focus group discussions in Iquitos (Phase 1).

| Issues | Main topics that emerged from focus group participants |
| --- | --- |
| Aesthetics | Mosquitoes are attracted to the dark, so traps should be dark. Traps should be size of an office trash can (~30 cm in height). |
| Concerns about Safety | Much concern regarding young child and pet safety, coupled with concern for what children and pets might do to the trap. Discussed possibility of trap being placed high (not preferred entomologically because *Aedes aegypti* prefer resting in locations up to 1 meter off ground), within a safety mesh (e.g. chicken wire), or with a solid lid that could not be easily opened. |
| Durability | Unanimous agreement that traps should be made of durable material requiring an initial investment and then refilled regularly (vs. requiring routine purchasing and creating more trash – even if biodegradable). Disposable traps would increase trash in the environment. |
| Maintenance | Unanimous agreement that someone in home would be willing to carry out maintenance activities. |
