# Supplementary material for: Design and Testing of Novel Lethal Ovitrap to Reduce Populations of Aedes Mosquitoes: Community-Based Participatory Research between Industry, Academia and Communities in Peru and Thailand
Source: PLoS One. 2016 Aug 17;11(8):e0160386. doi: 10.1371/journal.pone.0160386 (PMC4988764; doi:10.1371/journal.pone.0160386)
Supplement: S2 Table — (DOCX) [file pone.0160386.s002.docx]

**S2 Table:** Summary of discussion points between research and design teams about initial 22 trap designs regarding features needed and wanted for the traps (Phase 2).

| **Feature** | **Requirement** | **Comments** |  |
| --- | --- | --- | --- |
| **Life/ Durability** | Trap components shall be reusable for at least one year. Toxicants must last 90 days. | Testing has shown toxicants can last 90 days. |  |
| **Ecological impact** | Trap should be durable (vs. recyclable). | The refill for one trap model option consisted of a bowl with paper lid to be placed directly in traps – may create trash. |  |
| **End of Life** | Avoid breeding ground for illness carrying mosquitoes. | How will weather affect materials, biodegration, larvicides & adulticides? |  |
| **Maintenance** | Shall be easy to maintain accurately | Maintenance includes cleaning out debris, refilling with water, and recharging the larvicide and attractant? Small animals may drink water, or drown in trap. |  |
| **Recharge** | Easy to recharge every 90 days | Add beads and larvicide; replace adulticide net |  |
| **Color** | Trap shall be black or red | Research showed black was acceptable.  Red was tested and found to be more visible in low light conditions. |  |
| **Light-Shield** | Inside of trap must be dark | Roof creates shade in trap |  |
| **Stability** | Must be stable, able to withstand tipping from weather or normal interaction | Users indicated that stability was very important to them in the research. Must appear ´rugged´ for them to place outside. |  |
| **Out of Box Set-Up** | Shall have minimal and easy to perform out of box set-up without tools | Need to understand user perceptions on interacting with the target product components |  |
| **Installation** | Must be easy to install. Encourage placement below 3 feet. | Must be safe from damage of typical things in outside environment. Availability of water in target locations to be considered. |  |
| **Indicator for proper functioning** | Trap must contain some dead mosquitoes | Screen above water will be sticky to retain mosquitoes |  |
| **Refill** | Indicate to user refill needed | Color change tab or a date sticker |  |
| **Water level indicator** | Shall have water level visible from exterior of trap |  |  |
| **Rain** | Needs roof to prevent rain from entering trap | Keep the bulk of the rain out, but provide drain holes as well |  |
| **Safety** | Must not allow children´s hands inside of trap | Avoid hazards, such as pinch points, suffocation, larvicide and adulticide exposure. |  |
| **Attractant Delivery** | Keep attractant filled beads moist for required life of the product | Assumption is that we are using the current formulation, not a waterless solution |  |
| **Adulticide delivery** | At least 6” (height) of adulticide exposure within trap | Must be minimal risk to user. |  |
| **Entryway to trap** | At least 2.5” from body of trap to roof | Ideal based on target product profile established during cage testing |  |
| **Manuf. Cost** | | ˂$3.00 | This is based on 3 month disposable trap |
| **Shipping** | | Optimized for easy shipping | Traps may be disassembled for shipping |
