# Supplementary material for: Design and Testing of Novel Lethal Ovitrap to Reduce Populations of Aedes Mosquitoes: Community-Based Participatory Research between Industry, Academia and Communities in Peru and Thailand
Source: PLoS One. 2016 Aug 17;11(8):e0160386. doi: 10.1371/journal.pone.0160386 (PMC4988764; doi:10.1371/journal.pone.0160386)
Supplement: S3 Table — (DOCX) [file pone.0160386.s003.docx]

**S3 Table:** Summary of nine focus group discussions regarding 6 trap models in Peru and Thailand (Phase 3).

| **Issues** | **Summary of discussion** |
| --- | --- |
| **Child and pet safety** | In both sites, many questions regarding the risk to children if they touched, played or even drank the liquid with larvicide; there were concerns about toxicity. Solutions discussed in both sites included placing traps in high places (i.e., shelves or hanging on walls). In Lopburi, concern about whether electric fans might spread the smell of toxicants from the trap, and whether this could be harmful to the children. Also, discussion in both sites that pets might try to drink the larvicide, or move or tip over traps. |
| **Size of trap cover** | All participants felt the trap covers were too small or weak to keep out the rain and that this would influence their choice for type of trap chosen. They recommended increasing the perimeter of the trap cover, using stronger materials for the cover, and ensuring that covers could be secured. Additionally, in Lopburi, some participants were unsatisfied with other aspects of the cover: 1) they felt it had to be designed so that it could be removed more easily for cleaning, 2) they felt that the space between the cover and trap had to be larger so that more mosquitoes could fly in, and 3) they felt that there should be a smaller cover for indoor traps compared to outdoor traps for aesthetic reasons. |
| **Trap cover secured to base** | Some models had covers that did not securely attach to the base: covers should consider strong wind or curious fingers; they need a solid attachment, but also one that allows for easy checking and access. |
| **Space between cover and base** | The space needs to be large enough to let *Ae. aegypti* in, but keep pets and curious fingers out. |
| **Stability** | The majority of participants felt that the traps did not have enough stability for either indoor or outdoor use. They recommended using stronger materials, changing the shapes from rounded to squared, and adding a heavier base for a lower center of gravity. While Iquitos participants discussed trap options that could be staked to the ground or walls for stability due to their dirt floors and possible flooding, in Lopburi, many of the women did not like the design of the traps that were staked to the ground because of the requirement for extra tools and efforts to place them. Models "I" and "H” were considered particularly “unstable” looking in both sites. |
| **Rain outlet** | Regardless of trap cover, it was agreed that traps might fill up with rain so a little drainage hole to release excess water was needed. Another option was to be able to easily check the water level: model “I” had a transparent “window” that was highly valued in both sites, both for its practicality in allowing them to check for water levels (Iquitos and Lopburi) and because it made the traps look modern (Lopburi). |
| **Biodegradable/ ecological impact/ durability** | Some trap options came with easy to use interior packages for beads/attractants (i.e., lift lid and place in trap), but people in both sites discussed minimizing trash and ecological impact. All preferred traps that were made of durable materials and that were completely reusable for ecological reasons; this would also allow them to spend less money in the long run. In Iquitos and Lopburi, they expressed preference for changing parts at minimum every 6 months or 1 year, but in Lopburi they felt it would be acceptable if they had to change out parts every three months as long as the replacements were not expensive and widely available. In Lopburi, participants stated they would wrap them when in the off-season so that they last longer. In Iquitos, participants agreed on the benefits of using biodegradable materials, as long as it did not raise significantly price of a trap or its parts. |
| **Aesthetics** | In both study sites, participants agreed that black and dark colors in general attract mosquitoes. All FGD participants in Iquitos agreed with the choice of a black trap because they thought this color is “*most attractive to mosquitoes*”, and felt that including another color would be for decorative purposes only. In Lopburi, young female FGD participants preferred bright colors like red or blue because black inside the home is associated with bad luck; men and older women in Lopburi prioritized the functionality of the traps over the color. In both sites, most participants focused on functionality over aesthetics; for example, when asked which model they would prefer in their homes and why, participants started by talking about which model seemed durable and stable. However, in both sites, participants also expressed preference for different models based on whether these would be placed within the house, in which case they may select one that is more decorative, compared to the ones that might be placed outside which had to be more durable. In Lopburi, there was some discussion about one trap model that looked like a lamp and might be hung as decoration. |
| **Expected placement** | Placement preference was clearly associated with participants’ impression of stability, size, and type of trap cover. For more stable traps, participants chose places like living rooms; for less stable traps, the places selected were hidden from view. Gender differences were more apparent in the responses in the Lopburi FGD, with men preferring traps that were movable, so that these could be moved around the home where people are gathered or even taken to the rice fields, whereas the women discussed specific models in specific locations based on aesthetics (i.e., model J on the wall for decoration) or durability (i.e., model P outdoors because it looks stronger). In the Iquitos FGD, size of the trap mattered for placement: people talked about placing the traps under their beds if these were small, and this would allow them to “sleep deeply”. |
| **Maintenance** | In both sites, checking the water every week was generally considered acceptable, and a task that would likely be carried out by women (although in Lopburi they suggested that even children could help their parents with this). Some participants felt that some people would forget to do weekly water checks and total trap cleaning every 2 months: *“not everyone has the same level of responsibility or good memory to remember doing it.”* When asked what would help to remind them to check the water and clean out the trap, participants suggested placing the trap in visible places. In Lopburi, there was also concern about the efficacy of the trap if they did accidentally forget to refill the water. |
| **Costs** | Participants preferred purchasing traps in health centers, or at veterinarian clinics, stores, supermarkets or popular stores in the city, despite the fact that in Iquitos, most buy their vector-control products at the large market or from a regular vendor. In Iquitos, most participants described anything between US$3-7 as a “manageable” price for the trap, and for spare parts, they thought US$1-2 would be acceptable, depending on the size and type of part being purchased. In Lopburi, participants gave a maximum price of US$10 for the whole trap. They differentiated costs based on the materials used for the traps, for example, feeling that one trap (model J) should be more expensive because it had metal. Other designs could cost US$1-3, but perceived that the costs might be high because the traps would be imported. In Lopburi, participants felt that the government should play a role in subsidizing the trap costs or even providing them for free, considering that these could be more effective than fumigations. They added that the disposable parts of the traps should be available at low cost and easily accessible, whether at a local market or from government officers. |
| **Overall suggestions for improvement** | Participants recommended having smaller traps or having the same trap in different sizes so they could choose the right one according to placement location. In Iquitos, the use of a screen, netting materials or sponges to prevent dead mosquitoes from accumulating in the trap was recommended for all traps that did not have it. In Lopburi, people described they would want to clean the trap regularly to keep the inner part of the trap free of dead mosquitoes and insects. In both sites, people were pleased that they were asked for their perspectives, and volunteered to participate again. |
