# Supplementary material for: Design and Testing of Novel Lethal Ovitrap to Reduce Populations of Aedes Mosquitoes: Community-Based Participatory Research between Industry, Academia and Communities in Peru and Thailand
Source: PLoS One. 2016 Aug 17;11(8):e0160386. doi: 10.1371/journal.pone.0160386 (PMC4988764; doi:10.1371/journal.pone.0160386)
Supplement: S4 Table — (DOCX) [file pone.0160386.s004.docx]

**S4 Table:** Summary of four focus group discussions with community members in Iquitos and Lopburi discussing four trap models (Phase 5).

| **Characteristics that the traps must have** | In Iquitos, the absolute necessary components for the trap were: effectiveness, durability, easy to use, safe and stable, reminder piece (i.e., a tag or sticker or small window to see into trap). Same “must-haves” were desired in Lopburi, with exception to reminder piece. A nice design was positive in both settings, but not necessary. |
| --- | --- |
| **Size** | Mock up trap sizes (30 cm tall) presented were acceptable. However, there was still a described preference for smaller traps indoors, and larger traps with a strong base for the outdoors. |
| **Stability and placement** | In Iquitos, much concern was expressed about trap stability: on rainy and windy days, people still felt they would need to bring traps inside or they might tip over. Models J and H were considered the most stable, and hence most preferred for outside placement.  In Lopburi, model “J” was the most popular one for both indoor and outdoor use – people remarked on its strong wooden base and the rod made of steel, but questioned the durability of the compressible part which did not look strong enough for the heavy rains and strong wind of the region. Model “H” was the second choice for indoor use. Participants liked and commented on the strong and wide base of model “P”, as well as its reminder tag. They suggested that it would be good for both indoor and outdoor use. Model “M” was considered good only for indoor use. There were two concerns about this model: it looked like it would be a bit inconvenient to remove the lid, and there was the perception that the rods under the roof might not attract mosquitoes to fly in to the trap. |
| **Durability** | Durability was associated with stability: it was perceived that more stable traps were made with stronger materials, and hence would be more durable. In both sites, all parts made of steel and thick plastic were considered durable. Wooden parts were perceived as less durable in Iquitos, especially outdoors where these could erode with exposure to the elements. |
| **Withstanding outdoor elements** | The intensity of rain and wind were always mentioned as concerns in both sites. In Iquitos, beyond issues of rain getting into traps from the top, rain implies having high levels of water and mud in the backyard, and people stated that traps using stakes should have long and thick stakes to prevent them from falling down. For those without stakes, people recommended that traps be placed on small tables to keep them safe. To withstand strong wind, people recommended taking traps inside the home or placing them in areas where wind could not affect them. In Lopburi, people felt that the traps with strong base and holders would be good for outdoor use. Model 'J' looked strongest to them, but they were not sure about its use outdoors if it was very windy or raining heavily. |
| **Maintenance** | Checking the water levels every week and replacing the spare parts every 3 months was considered feasible by participants to remember, especially if they had reminder tags or stickers. |
| **Refilling of traps** | All traps were considered easy to refill. In Iquitos, filling water through a hole in the base (model P) and removing the bowl (model J) were most valued; the first because it was considered most practical (3 FGD) and the latter because it was considered slightly safer (1 FGD). In Lopburi, they felt it would not be difficult to remove parts to refill any of the traps with water. |
| **Reminders to refill: tags** | Two tag options were presented – one with an expiration date and one that changed color when it was time to change. In both sites, the tag that changes its color was preferred because it was more novel, attractive, and visible than other tags. In Lopburi they also discussed how helpful this would be, particularly the older groups. |
| **Continue to use other control methods?** | The majority stated they would continue using other methods to control mosquitoes while also using the trap. The message that traps could work just for mosquitoes that transmit dengue seemed well understood by most: *“if this trap is just for dengue then we need to continue using the other methods for mosquitoes that are active during night and other insects.”* In Lopburi, they stated they would continue using their regular vector control methods due to the uncertainty about the effectiveness, cost, and availability of the traps. |
| **Ranking traps based on perceived cost** | Groups were asked to rank, from least to most expensive, the traps. Two groups ranted them as M-H-P-J, while the other 2 ranked them H-M-P-J. Expected trap costs ranged from US $3.5 (model M), to $5 (model H), up to $25 (model J) based on the perceived quality and quantity of materials. For example, model ‘J’ was considered the most expensive because it has more parts and some are made of steel.  In Lopburi, expected trap costs were US$3-5. Like in Iquitos, it was expected that model J would be the most expensive and that one might pay up to US$10 for it, whereas the model H would be the be most inexpensive. |
| **Safety** | Model "P” was generally considered safest because it was more stable and less likely to fall if bumped into by children and animals. Model "J" was voted second most safe, again, because of its stability. |
| **Top choice** | The top choice in 3 FGD was model “P”; “J” was second. In the other FG, model “P” was followed by “M”. In the 4 FGD in Lopburi, the top choice was model “P”, followed by model “M” which was preferred for indoor use and considered “safe enough”. |
